# Incidence rates of post-pancreatectomy complications are similar between octogenarians and non-octogenarians and decrease after minimally invasive pancreatic surgery : a retrospective cohort study

**DOI:** 10.1186/s12893-025-03184-1

**Published:** 2025-10-03

**Authors:** Ryo Saito, Hidetake Amemiya, Wataru Izumo, Yuuki Nakata, Takashi Nakayama, Kazunori Takahashi, Suguru Maruyama, Koichi Takiguchi, Katsutoshi Shoda, Kensuke Shiraishi, Shinji Furuya, Yoshihiko Kawaguchi, Hiromichi Kawaida, Daisuke Ichikawa

**Affiliations:** Faculty of Medicine, First Department of Surgery, University of Yamanashi, 1110 Shimokato, Chuo, Yamanashi, 4093898 Japan

**Keywords:** Pancreatectomy, Complication, Laparotomy, Octogenarians, Minimally invasive surgery

## Abstract

**Background:**

This study investigated the incidence of postoperative complications following pancreatectomy in octogenarians.

**Methods:**

This study included 291 patients who underwent pancreatic surgery (pancreatoduodenectomy [PD] or distal pancreatectomy [DP]) between 2019 and 2024 in a Japanese University Hospital. Perioperative outcomes were compared between octogenarians and non-octogenarians. The primary outcomes included the risk factors (including age) and incidence rates for postoperative complications. In addition, 1:3 propensity score matching (PSM) was conducted with standardized patient and perioperative factors, and the incidence of postoperative complications was compared. Finally, the clinical characteristics of octogenarians and non-octogenarians in open surgery and minimally invasive pancreatic surgery (MIPS) were compared.

**Results:**

The median age was 82.0 and 71.0 years for octogenarians (*n* = 33) and non-octogenarians (*n* = 258). We found that a high body mass index (BMI) value, PD, laparotomy, high intraoperative blood loss (IBL) (≥ 320mL), and long operative times were associated with the incidence of postoperative complications in univariate analysis, although patient age (octogenarians) was not significantly correlated. PSM showed that the incidence of postoperative complications was similar between octogenarians and non-octogenarians. MIPS led to similar or lower postoperative complication rates in octogenarians compared with non-octogenarians, and both rates were notably lower than those in laparotomy.

**Conclusion:**

Although this study was retrospective, single-center, and with small number of octogenarians, the incidence of postoperative complications after pancreatectomy might not be higher in octogenarians than in non-octogenarians, and it could be reduced with MIPS.

## Introduction

In recent years, the incidence of pancreatic diseases, including pancreatic cancer, has been increasing [1]. Most pancreatic diseases require pancreatic resection, but pancreatectomy leads to a high incidence of postoperative complications [2]. The most common complication is postoperative pancreatic fistula (POPF), but if treatment is not effective, it can lead to postpancreatectomy hemorrhage from a pseudoaneurysm, which can be fatal [3]. In addition to POPF, there are various complications, including delayed gastric emptying, bile leakage (in the case of pancreatoduodenectomy [PD]), long-term nutritional disorders as well as diabetes and other complications [4]. We have previously reported the characteristics of complications after pancreatectomy in detail, and the incidence rate of postoperative complications with Clavien-Dindo (CD) classification grade 2 or higher was high, approximately 70% for PD and 40% for distal pancreatectomy (DP) [5]. It has also been shown that the occurrence of postoperative complications significantly extends the postoperative hospital stay (POHS). Long-term hospitalization may directly lead to an increase in the physical and financial burden on the patient and to a decrease in physical strength, activities of daily living (ADL), and performance status (PS) [6]. Therefore, it is necessary to accurately assess the perioperative risks for each patient and determine a suitable management plan individually. On the other hand, in the current perspective of an aging society, the number of patients with pancreatic diseases that require pancreatectomy, even among the octogenarians, has been increasing [7, 8]. In general, the greater the age of elderly patients, the more likely they are to have comorbidities and organ disorders, and the less likely they are to have overall surgical tolerance [9]. Thus, some elderly patients are deemed not amenable to surgery simply because of their age, while other elderly patients may experience a serious impairment of their ADL and/or PS due to postoperative complications, and other patients may even lose their lives [10]. In our modern society, where social conditions are changing dramatically, it is becoming increasingly difficult to accurately determine whether octogenarians may safely undergo pancreatic surgery.

Meanwhile, the era of minimally invasive surgery has arrived, even in pancreatic surgeries which have hitherto been considered highly invasive procedures. Accordingly, laparoscopic and robotic pancreatectomy are becoming more widespread, and favorable results have been reported [11]. The benefits of minimally invasive pancreatic surgery (MIPS) may be particularly notable in elderly patients with significantly reduced physical strength and surgical tolerance [12, 13]. In this study, we clarified the risk of incidence of postoperative complications following pancreatectomy in octogenarians and verified the advantages of MIPS. We hope that the results of this study will contribute to providing appropriate opportunities for safe and optimal pancreatectomy for octogenarians.

## Methods

### Patients

Patients who underwent PD or DP as typical pancreatic surgeries at the University of Yamanashi Hospital, Japan, between January 2019 and December 2024 were included for an analysis of complications resulting from surgical interventions for diseases of the pancreas, bile duct, duodenum and others. Patients who underwent simultaneous surgery in other organs were excluded from the present study. Patients who underwent central or total pancreatectomy, enucleation, bile or digestive tract bypass surgery and other atypical surgery were also excluded.

### Ethics approval and consent to participate

This study was approved by the Ethics Committee of the University of Yamanashi (Ethical Approval Number: 2044) and was conducted in accordance with the ethical standards of the Declaration of Helsinki and its later amendments [14]. The requirement for informed consent to participate was waived by the Ethics Committee because this was a retrospective observational study using anonymized clinical data [15].

### Comparison between octogenarians and non-octogenarians

Patients analyzed in this study were classified into octogenarians aged 80 years or older and non-octogenarians aged under 80 years. Postoperative complications were evaluated following CD classification, and CD grade ≥ 3 was defined as a clinically significant complication and set as the primary outcome of this study, to be compared between the two patient groups [16]. Secondary outcomes included the occurrence of POPF and length of POHS. Clinical and perioperative factors were evaluated using physical findings such as age, sex, and body mass index (BMI), as well as American Society of Anesthesiologists Physical Status (ASA-PS) scores to evaluate systemic comorbidities, with an ASA-PS score of 3 or higher defined as poor physical condition, i.e., high-risk due to comorbidities [17]. This study included patients with various diseases exhibiting clear indications for pancreatic resection, such as pancreatic ductal adenocarcinoma (PDAC), pancreatic neuroendocrine tumor (pNET), intraductal papillary mucinous neoplasm, distal bile duct cancer, duodenal ampullary tumor, and other duodenal tumors.

### Surgical procedure and perioperative factors

To equalize the perioperative outcomes, patients who underwent PD or DP were analyzed in this study. PD included subtotal stomach preserved-PD and pylorus preserved-PD. Patients admitted for treatment in the earlier half of the study underwent either sub-total stomach preserved-PD or pylorus preserved-PD while patients admitted in the latter half of the study underwent pylorus preserved-PD. The reconstruction method employed was the modified Child surgery technique, and pancreatojejunostomy, cholangiojejunostomy, and gastro- or duodenojejunostomy were carried out. The majority of patients with PDAC or pNET underwent standard regional lymphadenectomy, and limited lymphadenectomy was performed for benign diseases. For PD, open surgery was performed in the early phase of this study; however, laparoscopic/robotic surgery has been introduced recently, primarily for low-grade malignant tumors. Similarly for DP, where laparoscopic or open surgery had been performed, robotic surgery is indicated for almost all cases nowadays. The background information for patients included age, sex, BMI and other parameters. According to the 2022 Japanese Clinical Practice Guidelines for Pancreatic Cancer, neoadjuvant chemotherapy (NAC) is widely indicated for PDAC, except for patients with tumors without a preoperative pathological diagnosis, or those who were not indicated due to their general condition [18]. NAC is primarily indicated for gemcitabine/S-1 therapy for resectable pancreatic cancer and gemcitabine/nab-paclitaxel therapy for borderline resectable pancreatic cancer. For the purpose of this study, POPF was defined as Grade B or higher, as described by the International Study Group for Pancreatic Surgery [19]. The number of days from surgery to discharge was defined as POHS. Postoperative complications were assessed comprehensively based on imaging evaluations such as computed tomography, blood tests, drain evaluation, and physical examination findings. Surgical sight infections (SSI) was defined according to the CDC/NHSN criteria (2023 version), which classifies infections as superficial incisional, deep incisional, or organ/space. Organ-space SSI included intra-abdominal abscesses and clinically relevant postoperative pancreatic fistulas (Grade B/C) when associated with purulent drainage or positive cultures. Cutoff values for continuous variables in perioperative factors were determined by plotting receiver operating characteristic curves, concerning incidence of postoperative complications with CD grade ≥ 3, using the Youden index [20].

### Analytic procedure

First, we compared patient backgrounds and perioperative factors between octogenarians and non-octogenarians, including the incidence of postoperative complications, POPF, and the duration of POHS. Next, we clarified the Risk factors for incidence of postoperative complications using univariate followed by multivariate analysis. Then, we performed a 1:3 propensity score matching (PSM) with a caliper value of 0.2, to extract patients and compare the incidence of postoperative complications based on a similar background between octogenarians and non-octogenarians. Finally, we divided the patients into open surgery and MIPS groups, compared the characteristics of those in each group, and verified the conditions for safe pancreatic surgery in the octogenarians.

### Statistics

Comparative analyses among the patient groups were performed using the chi-square test or Fisher’s exact test for nominal variables, as appropriate. The normality of continuous variables was assessed using the Shapiro–Wilk test, and statistically analyzed using Mann-Whitney U tests. Logistic regression analysis was used to calculate odds ratios for each factor concerning incidence of postoperative complications and for multivariate analysis with statistically significant variables having *p*-values < 0.05 in univariate analysis. Statistical significance was set at *p* < 0.05. All statistical analyses were performed using EZR software (Saitama Medical Center, Jichi Medical University, Saitama, Japan), which is a graphical user interface for R software (R Foundation for Statistical Computing, Vienna, Austria) [21].

## Results

### Comparison of clinicopathological characteristics between octogenarians and non-octogenarians

**Fig. 1:**
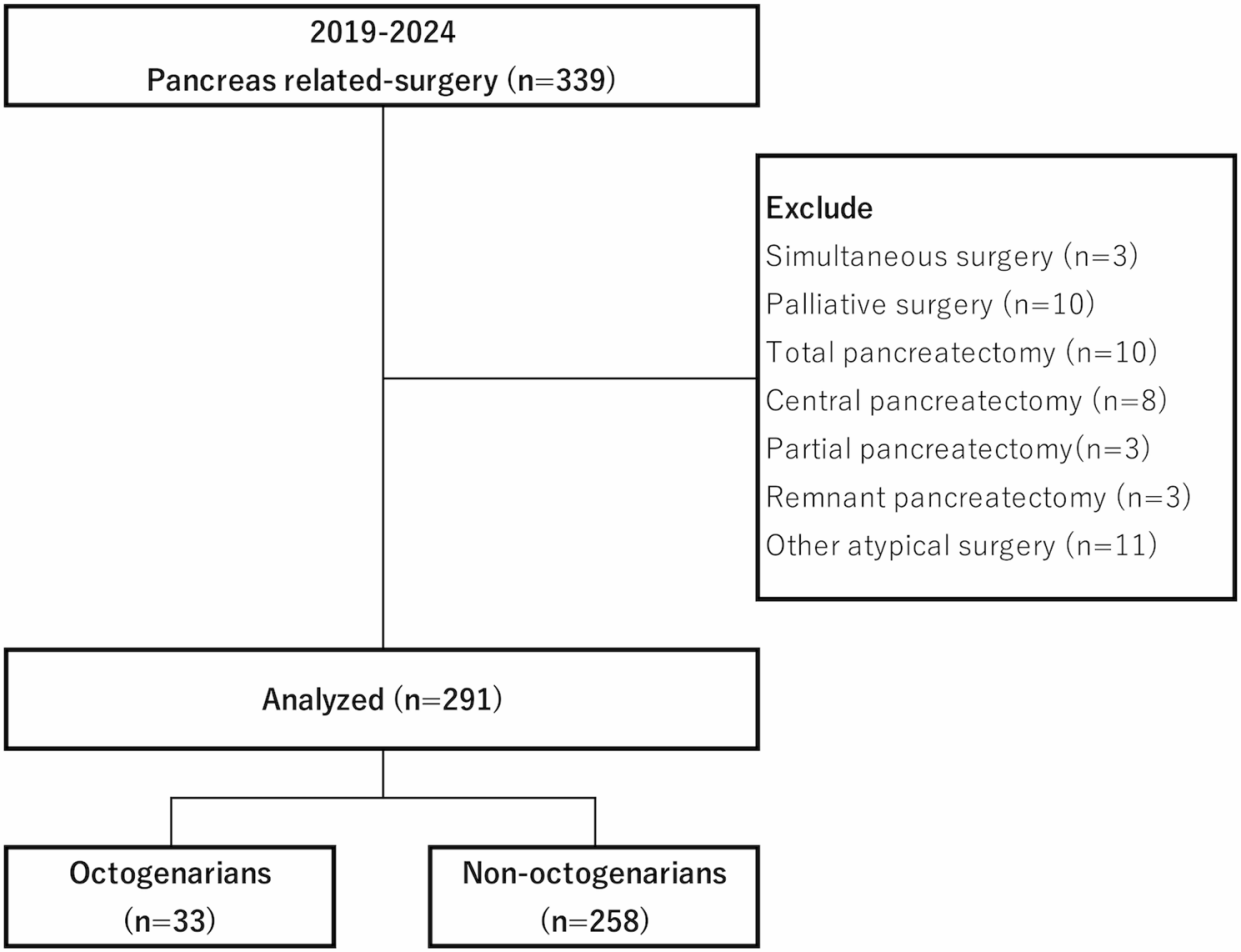
Patients flow diagram. Total of 339 people underwent pancreatic-related surgery during 2019-2024. Patients who underwent simultaneous surgery in other organs, central or total pancreatectomy, enucleation, bile or digestive tract bypass surgery and other atypical surgery were also excluded. Finally, 291 patients were included in the study. Patients were classified into octogenarians aged 80 years or older and non-octogenarians aged under 80 years

Of the total 339 people who underwent pancreatic-related surgery during this period, 291 patients were included in the study (Fig. 1). Of the 291 patients analyzed, 33 were classified as octogenarians and 258 as non-octogenarians. Table 1 shows a comparison of clinicopathological characteristics between octogenarians and non-octogenarians. The median age of patients in the octogenarian group was 82.0 years and that of the non-octogenarian group was 71.0 years. Compared with the non-octogenarian group, the octogenarian group had a higher ASA-PS score, but the sex composition and BMI values were similar in the two groups. There were also no differences in the proportion of PADC, surgical method (PD or DP), or surgical approach (laparotomy, laparoscopy, or robot-assisted surgery). This study included two patients with ASA-PS4. One was an octogenarian, and one was a non-octogenarian. Since the subjects of this study were patients who underwent radical and conventional pancreatic resection, these patients also underwent conventional pancreatic resection (PD for the former and DP for the latter). These patients underwent surgery following careful multidisciplinary evaluation, and both had localized disease without major comorbidities besides the anesthetic risk factors. Their surgical indications were considered curative.

**Table 1:** Clinicopathological features of octogenarians and non-octogenarians

Factor	Group	Octogenarians	Non-octogenarians	*p*-value
		*n* = 33 (%)	*n* = 258 (%)	
Age (y.o.)		82.0 [80–87]	71.0 [19–79]	< 0.001
Sex	Men	19 (57.6)	154 (59.7)	0.852
ASA-PS	≥ 3	16 (48.5)	74 (28.7)	0.027
BMI (kg/m^2^)		21.7 [18.0-32.9]	22.2 [13.1–33.2]	0.511
Disease	PDAC	18 (54.5)	123 (47.7)	0.467
NAC	Performed	7 (21.2)	87 (33.7)	0.306
Procedure	PD	19 (57.6)	142 (55.0)	0.854
	DP	14 (42.4)	116 (45.0)	
Surgical approach	Open	24 (72.7)	155 (60.1)	0.383
	Lap	7 (21.2)	69 (26.7)	
	Robot	2 (6.1)	34 (13.2)	
IBL (mL)		472.0 [3-2386]	357.5 [1-5878]	0.101
OPT (min)		460.0 [210–789]	469.0 [182–834]	0.513
Postoperative complication	≥ CD 3	7 (21.2)	49 (19.0)	0.815
POPF	Grade B/C	9 (27.3)	63 (24.4)	0.675
SSI	Present	10 (30.3)	75 (29.1)	0.842
POHS (day)		18.0 [8–88]	17.0 [6-114]	0.657

*ASA-PS* American society of anesthesiologists physical status, *BMI* body mass index, *NAC* neo-adjuvant chemotherapy, *IBL* intraoperative blood loss, *OPT*, operative time, *SSI* surgical site infection, *POPF* postoperative pancreatic fistula, *POHS* postoperative hospital stay, *PDAC* pancreatic ductal adenocarcinoma, *PD* pancreatoduodenectomy, *DP* distal pancreatectomy, *lap* laparoscopy, *CD* Clavien-Dindo classification, *y.o.* year old

The surgical indication was curative at the time of operation based on preoperative imaging and tumor markers. The incidence of postoperative complications, duration of POHS, and POPF were similar as well. In this cohort, there were no deaths within 30 days after surgery or in-hospital deaths. Two patients died within 90 days after surgery, both in the non-octogenarian group. One patient died due to progression of another cancer, and the other died due to accidental drowning. In this patient, both cancers were deemed curative at the time of surgery. However, after pancreatic resection, the other cancer progressed faster than expected, and despite aggressive treatment, the patient died due to the progression of the other cancer.

All 33 octogenarian patients had some sort of systemic comorbidities. Twelve had a history of cancer treatment. There were many patients of severe comorbidities, with eight patients having cardiovascular complications and six having cerebrovascular complications. In addition, many patients had comorbidities associated with decreased systemic function, such as hypertension (22 patients), diabetes (9 patients), and renal dysfunction (12 patients).

### Factors associated with the incidence of postoperative complications

Table 2 shows the factors associated with the incidence of postoperative complications for patients with a CD grade ≥ 3. Univariate analysis showed that high BMI values, PD, laparotomy approach, high intraoperative blood loss (IBL), and long operative times (OPT) were significant factors associated with the incidence of postoperative complications. In addition, the male sex and non-PDAC status tended to be associated with the incidence of postoperative complications but not to the extent of statistical significance, while octogenarians was not associated with the incidence of postoperative complications. A multivariate analysis using factors that showed statistical significance (*p* < 0.05) in univariate analysis showed that high BMI values and high IBL were independently significant factors associated with the incidence of postoperative complications.

**Table 2:** Univariate and multivariate analyses for incidence of postoperative complications

Factor	*n*	Univariate			Multivariate		
		Odds ratio	95% C.I.	*p*-value	Odds ratio	95% C.I.	*p*-value
Octogenarians	33	1.15	0.471-2.80	0.815			
Sex (Men)	173	1.73	0.925–3.23	0.096			
ASA-PS (≥ 3)	90	1.19	0.639-2.20	0.630			
BMI (≥ 23.5 kg/m2)	108	3.06	1.68–5.57	< 0.001	2.41	1.29–4.53	0.006
Disease (Non-PDAC)	150	1.74	0.952–3.16	0.075			
Procedure (PD)	171	2.12	1.14–3.97	0.017	1.03	0.415–2.54	0.954
Surgical approach (Open)	179	2.15	1.11–4.35	0.037	1.37	0.425-4.40	0.599
IBL (≥ 320mL)	120	4.03	2.13–7.61	< 0.001	3.99	1.25–12.7	0.019
OPT (≥ 496 min)	152	2.75	1.46–5.17	0.002	1.55	0.767–3.14	0.221

*ASA-PS* American Society of Anesthesiologists Physical Status, *BMI* body mass index, *PDAC* pancreatic ductal adenocarcinoma, *PD* pancreatoduodenectomy, *IBL* intraoperative blood loss, *OPT* operative time, *C.I* confidence interval

### Propensity score-matched analysis for incidence of postoperative complications

The patient background related to the incidence of postoperative complications differs between the octogenarians and non-octogenarians: hence, we undertook PSM analysis to adjust for this bias. A 1:3 PSM was conducted and a total of 128 patients were extracted: 32 patients in the octogenarians and 96 in the non-octogenarians. The results of PSM revealed that the incidence of postoperative complications was similar in the two patient groups, 21.9% both in the octogenarians and non-octogenarians (*p* > 0.999) (Table 3). There were no significant differences in the POPF (25.0% vs. 29.2%, *p* = 0.821) or POHS (18.5 vs. 19.0, *p* = 0.775) values as well.

**Table 3:** Comparison of perioperative factors between octogenarians and non-octogenarians after PSM

Factor	Group	Octogenarians	Non-octogenarians	*p*-value
		*n* = 32 (%)	*n* = 96 (%)	
Age (y.o.)		82.0 [80–87]	70.0 [19–79]	< 0.001
Sex	Men	18 (56.2)	61 (63.5)	0.531
ASA-PS	≥ 3	16 (50.0)	30 (31.2)	0.088
BMI (kg/m^2^)		21.6 [18.0-32.9]	22.5 [13.1–31.9]	0.917
Disease	PDAC	18 (56.2)	51 (53.1)	0.839
Procedure	PD	19 (59.4)	57 (59.4)	> 0.999
	DP	13 (40.6)	39 (40.6)	
Surgical approach	Open	23 (71.9)	69 (71.9)	0.943
	Lap	7 (21.9)	19 (19.8)	
	Robot	2 (6.2)	8 (8.3)	
IBL (mL)		467.0 [3-2386]	477.0 [6-5878]	0.530
OPT (min)		454.5 [210–789]	472.0 [182–749]	0.395
Postoperative complication	≥ CD 3	7 (21.9)	21 (21.9)	> 0.999
POPF	Grade B/C	8 (25.0)	28 (29.2)	0.821
POHS (day)		18.5 [8–88]	19.0 [8-114]	0.775

*PSM* propensity score matching, *ASA-PS* American Society of Anesthesiologists Physical Status, *BMI*, body mass index, *IBL* intraoperative blood loss, *OPT* operative time, *POPF* postoperative pancreatic fistula, *POHS* postoperative hospital stay, *PDAC* pancreatic ductal adenocarcinoma, *PD* pancreatoduodenectomy, *DP* distal pancreatectomy, *Lap* laparoscopy, *CD* Clavien-Dindo classification, *y.o*. year old

### Minimally invasive surgery reduces the risk of postoperative complication occurrence in octogenarians

Finally, we compared the octogenarians and non-octogenarians in open surgery and MIPS, respectively. As a result, the incidence rates of postoperative complications trended slightly higher in the octogenarians compared with the non-octogenarians for open surgery (29.2% vs. 22.6%, respectively; *p* = 0.450) but lower in the octogenarians compared with the non-octogenarians for MIPS (0.0% vs. 13.6%, respectively; *p* = 0.599), and both results were lower than those for open surgery (Table 4). In addition, we also found that in both patient groups, the duration of POHS was shorter with MIPS than with open surgery. These results suggest that the MIPS intervention for significantly octogenarians may reduce the incidence of postoperative complications and shorten POHS.

**Table 4:** Comparison of perioperative factors between MIPS and open surgery

Factor	Group	Open			MIPS		
		Octogenarians	Non-octogenarians		Octogenarians	Non-octogenarians	
		*n* = 24 (%)	*n* = 155 (%)	*p*-value	*n* = 9 (%)	*n* = 103 (%)	*p*-value
Age (y.o.)		82.5 [80–87]	71.0 [31–79]	< 0.001	82 [80–87]	68 [19–79]	< 0.001
Sex	Men	15 (62.5)	99 (63.9)	> 0.999	4 (44.4)	55 (53.4)	0.733
ASA-PS	≥ 3	13 (54.2)	57 (36.8)	0.119	3 (33.3)	17 (16.5)	0.200
BMI (kg/m^2^)		23.4 [18.0-32.9]	22.2 [13.1–32.0]	0.136	21.3 [19.2–24.5]	22.3 [17.7–33.2]	0.246
Disease	PDAC	12 (50.0)	84 (54.2)	0.827	6 (66.7)	39 (37.9)	0.153
Procedure	PD	18 (75.0)	126 (81.3)	0.579	1 (11.1)	16 (15.5)	> 0.999
	DP	6 (25.0)	29 (18.7)		8 (88.9)	87 (84.5)	
Surgical approach	Open	24 (100.0)	155 (100.0)	NA			0.716
	Lap				7 (77.8)	69 (67.0)	
	Robot				2 (22.2)	34 (33.0)	
IBL (mL)		678.0 [81-2386]	616.0 [84-5878]	0.658	254.0 [3-806]	107.0 [1-1612]	0.304
OPT (min)		501.5 [210–789]	497.0 [220–834]	0.409	427.0 [253–524]	421.0 [182–785]	0.593
Postoperative complication	≥ CD 3	7 (29.2)	35 (22.6)	0.450	0 (0.0)	14 (13.6)	0.599
POPF	Grade B/C	8 (33.3)	44 (28.4)	0.633	1 (11.1)	19 (18.4)	> 0.999
POHS (day)		19.0 [9–88]	24.0 [8-114]	0.548	11.0 [8–27]	11.0 [6-105]	0.867

*MIPS* minimally invasive pancreatic surgery, *ASA-PS* American Society of Anesthesiologists Physical Status, *BMI* body mass index, *IBL* intraoperative blood loss, *OPT* operative time, *POPF* postoperative pancreatic fistula, *POHS* postoperative hospital stay, *PDAC* pancreatic ductal adenocarcinoma, *PD* pancreatoduodenectomy, *DP *distal pancreatectomy, *Lap* laparoscopy, *CD* Clavien-Dindo classification, *NA*, not available, *y.o.* year old

## Discussion

This study was conducted to clarify the risk of incidence of postoperative complications for octogenarians in the current era of MIPS. First, we showed that octogenarians had higher ASA-PS scores but there was no difference in the incidence of postoperative complications, POPF, or the duration of POHS. Next, we demonstrated that high BMI values, PD, open surgery, high IBL, and long OPT values were risk factors for the incidence of postoperative complications, while age (octogenarians) was not. Even after undertaking PSM, the incidence of postoperative complications, POPF, and duration of POHS values were similar among the octogenarians and non-octogenarians. Finally, we demonstrated that MIPS may be useful in reducing the incidence of postoperative complications in octogenarians, as well as non-octogenarians, and in improving short-term postoperative outcomes, such as shortening POHS.

In this study, high BMI values and high IBL were identified as factors especially related to the incidence of postoperative complications. When comparing PD and DP cases, it is plausible that PD involves more complex reconstructive procedures and is more invasive, making it more likely to cause postoperative complications [5]. High BMI values have previously been reported to be associated with the incidence of postoperative complications, and in pancreatic surgery, BMI has been reported to be associated with an increase in POPF, postoperative complications, and high IBL [22]. Although we found no differences in BMI values between the octogenarians and non-octogenarians in this study (21.7 vs. 22.2, *p* = 0.511), there are a certain number of patients with obesity in both the octogenarians and non-octogenarians. Therefore, attention should be paid to the occurrence of postoperative complications for such patients with obesity even in the octogenarians.

Conversely, it has been reported recently that high IBL is involved in POPF [23]. This does not simply reflect the surgical skills of surgeons but also indicates that the systemic invasion caused by excess IBL has a profound impact on the postoperative recovery process and may result in the incidence of postoperative complications [24]. In fact, this study also showed that high IBL has a stronger impact on the incidence of postoperative complications than operative time (Odds Ratio [OR] 3.99, *p* = 0.019 vs. OR 1.55, *p* = 0.221, as shown in Table 2). In general, MIPS has been shown to notably control IBL, although it extends the operative time [25]. It is believed that IBL control by MIPS contributes to the suppression of incidence rates for postoperative complications. In addition, we have previously reported from clinical and basic experimental data that when both high IBL and POPF conditions were met in pancreatic cancer patients, loco-regional recurrence was increased [26]. In this way, controlling IBL and avoiding the incidence of postoperative complications may also contribute to improving the long-term prognosis of pancreatic cancer patients.

In recent years, the results of randomized controlled trials (RCTs) have been reported comparing the short-term outcomes of MIPS and open pancreatectomy. In the reports comparing robot PD (RPD) and open PD (OPD), there was no difference in the incidence of postoperative complications between RPD and OPD cases, and it was suggested that both can be performed safely as well [27, 28]. If the incidence of postoperative complications is the same, RPD may be preferable, especially for octogenarians, as it is less invasive and may avoid a decline in ADL/PS. However, the results of RCTs focused on the long-term outcomes of RPD for pancreatic cancer have not been reported, and this is an issue to be resolved in the future. On the other hand, the findings of an RCT comparing minimally invasive DP (MIDP) and open DP (ODP) for pancreatic cancer have been reported [29]. According to this report, the incidence of postoperative complications (CD grade ≥ 3) was similar in MIDP (including RDP of 26.5%) compared with ODP, and no significant differences were observed in the short-term outcomes. The long-term outcomes were also comparable, proving the non-inferiority of MIDP to ODP in the treatment of pancreatic cancer. Meanwhile, age was found to be an independent prognostic factor in multivariate analysis of overall survival, and the extent to which therapeutic intervention should be undertaken in significantly octogenarians remains an intractable issue. Previous reports have examined the effect of improving the prognosis of octogenarians with pancreatic cancer [30, 31]. However, it is very difficult to accurately evaluate the possibility of surgery and whether or not a patient can receive postoperative chemotherapy, and it is necessary to make a judgment on each individual case. In addition, the number of days of postoperative hospitalization and the rate of home discharge are given as indicators of the impact of postoperative QOL. There are reports that the number of days of postoperative hospitalization of octogenarians is equivalent to that of the non-elderly [32] or longer [33], and there are also reports that the rate of home discharge is low in the elderly [34]. In any case, it is certain that the octogenarians are more likely to experience a decline in QOL, and the suitability of surgery must be carefully judged. It is also inevitable to consider the surgical procedure, surgical content, and approach method to avoid a decline in QOL. In that sense, minimally invasive surgery may be very useful. As shown in the present study, postoperative complications seem to occur based on patient background and perioperative factors rather than age and octogenarians cannot be used as a reason to refrain from pancreatic surgery. It is important to accurately evaluate the risk of each individual patient and provide appropriate treatment, regardless of age. In addition, MIPS may significantly contribute to reducing the incidence of postoperative complications and may drastically expand the options for pancreatic disease treatment, particularly for octogenarians.

This study has some limitations. First, this was a retrospective study conducted at a single institution, and the number of cases was small. Additionally, selection bias may have played a role in determining the indications for pancreatic resection in octogenarians. Although these biases are unavoidable in this study design, they are important points that may affect the results. To resolve these limitations, we believe that detailed examination in a large-scale prospective study is essential. Nevertheless, an analysis based on a uniform patient background after PSM-based patient extraction showed no differences in short-term outcomes including the incidence of postoperative complications, POPF, and duration of POHS between octogenarians and non-octogenarians. The results of this study should be notably useful in considering the indications for pancreatic surgery for octogenarians.

In conclusion, octogenarian patients undergoing pancreatectomy experience postoperative outcomes comparable to younger individuals, especially when minimally invasive techniques are applied. These findings support the potential utility of MIPS in selected octogenarians.

## Data Availability

The datasets generated and/or analysed during the current study are available from the corresponding author on reasonable request.

## Abbreviations

PD: Pancreatoduodenectomy
DP: Distal pancreatectomy
PSM: Propensity score matching
MIPS: Minimally invasive pancreatic surgery
BMI: Body mass index
IBL: Intraoperative blood loss
POPF: Postoperative pancreatic fistula
CD: Clavien-Dindo
POHS: Postoperative hospital stay
ADL: Activities of daily life
PS: Performance status
ASA-PS: American Society of Anesthesiologists Physical Status
PDAC: Pancreatic ductal adenocarcinoma
pNET: Pancreatic neuroendocrine tumor
NAC: Neoadjuvant chemotherapy
OPT: Operative time
OR: Odds ratio
RPD: Robot pancreatoduodenectomy
OPD: Open pancreatoduodenectomy
MIDP: Minimally invasive distal pancreatectomy
ODP: Open distal pancreatectomy
